# Contaminated Stream Water as Source for *Escherichia coli* O157 Illness in Children

**DOI:** 10.3201/eid2307.170226

**Published:** 2017-07

**Authors:** William S. Probert, Glen M. Miller, Katya E. Ledin

**Affiliations:** Napa-Solano-Yolo-Marin County Public Health Laboratory, Fairfield, California, USA

**Keywords:** Shiga toxin, *Escherichia coli* O157, deer, water, bacteria, children, enteric infections, California, United States

## Abstract

In May 2016, an outbreak of Shiga toxin–producing *Escherichia coli* O157 infections occurred among children who had played in a stream flowing through a park. Analysis of *E. coli* isolates from the patients, stream water, and deer and coyote scat showed that feces from deer were the most likely source of contamination.

In the United States, recreational water is a relatively uncommon source of Shiga toxin–producing *Escherichia coli* (STEC) O157 outbreaks (*1*). We describe an outbreak of STEC O157 infections among children exposed to a contaminated stream in northern California, USA, and provide laboratory evidence establishing wildlife as the source of water contamination.

In May 2016, four cases of Shiga toxin (Stx) 1– and 2–producing *E. coli* O157 infection were reported to a local health department in northern California; investigation revealed a common source of exposure. The case-patients, ranging in age from 1 to 3 years, had played in a stream adjacent to a children’s playground within a city park. Exposure of the case-patients to the stream occurred on 3 separate days spanning a 2-week period. Two case-patients are known to have ingested water while playing in the stream. Two case-patients were siblings. All case-patients had diarrhea and abdominal cramps; bloody diarrhea was reported for 3. One case-patient was hospitalized with hemolytic uremic syndrome.

The stream is a second-order waterway located in a northern California community of ≈7,500 residents. At the time of exposures, stream flow was <30 ft^3^/s. The land upstream is not used for agricultural activities such as livestock production. The community is serviced by a public sewer system; inspection of sewer lines indicated no breach to the system.

Water samples were collected from the exposure site 7 days after the last case-patient was exposed and weekly thereafter for 17 weeks; samples were tested quantitatively for fecal indicator organisms. Throughout the study period, all water samples exceeded recreational water quality limits for *E. coli* and enterococci levels (*2*). Water samples were also cultured for STEC isolation and PCR detection of *stx_1_* and *stx_2_* (*3*). Stx1- and Stx2-producing *E. coli* O157 were isolated from stream water each week for the first 4 weeks. Additionally, an Stx2-producing *E. coli* non-O157 strain was isolated from the stream in the first week of sampling. Enrichment broth cultures of water samples were also positive by PCR for *stx*1 and *stx*2 for the first 4 weeks of sampling. Thereafter, both *stx_1_* and *stx_2_*, or *stx_2_* only, were intermittently detected in enrichment broth cultures for 9 additional weeks.

In the absence of an obvious source (e.g., upstream agricultural operation or sewer leak), wildlife was considered as a possible contributor to water contamination. Thirteen fresh wildlife scat specimens were collected along the stream for STEC culture and PCR. Of the 13 scat specimens, 8 originated from deer, 2 from raccoon, and 1 each from coyote, turkey, and river otter. Six scat specimens (4 deer, 1 coyote, 1 river otter) were positive for *stx_1_* and *stx_2_* or for *stx_2_* by PCR (Technical Appendix). Stx1- and Stx2-producing *E. coli* O157 were isolated from deer scat and coyote scat. An Stx2-producing *E. coli* non-O157 strain was isolated from a deer scat specimen. The animal origin of the coyote and river otter scat specimens were definitively identified by partial DNA sequencing of mitochondrial cytochrome b (*4*).

To assess strain relatedness, we compared STEC O157 isolates from the case-patients, water, deer scat, and coyote scat by using pulsed-field gel electrophoresis (PFGE) and multilocus variable-number tandem-repeat analysis (MLVA) (*5*). PFGE patterns for *Xba*I-digested genomic DNA were highly similar among all isolates; only slight variations were found in the lower-sized bands (Figure). PFGE patterns for genomic DNA samples digested with *Bln*I also demonstrated a high degree of similarity (data not shown). Furthermore, MLVA profiles were identical for the case-patient, water, and deer scat isolates; only the coyote scat isolate differed from the main profile by 2 repeats at a single locus (VNTR_3).

**Figure:**
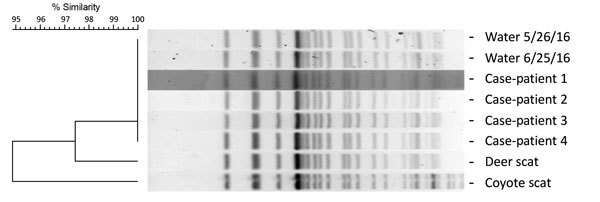
Pulsed-field gel electrophoresis (PFGE) analysis of Shiga toxin 1– and 2–producing *Escherichia coli* O157 isolates digested with *Xba*I. A dendrogram displaying PFGE pattern similarity is shown at left. The PFGE profiles for the case-patients and water isolates were identical and designated as pattern EXH01.0238 by PulseNet (https://www.cdc.gov/pulsenet/). The PFGE patterns for the deer and coyote scat isolates shared >95% similarity with pattern EXH01.0238. Dates on water samples indicate date of collection.

This study provides laboratory evidence linking STEC O157 infections with the ingestion of recreational water that was probably contaminated by wildlife scat. Wild ruminants, including deer and elk, are known carriers of STEC and have been connected to outbreaks of human infections (*6*–*9*). We detected STEC in 50% of deer scat specimens collected from the stream bank. One of these specimens, found 1.5 miles upstream of the exposure site, contained an *E. coli* O157 isolate that was highly similar by molecular subtyping to case-patient and water isolates. These findings support the likelihood that feces from deer carrying STEC were the source of water contamination or, at the very least, contributed to the persistence of STEC in the water. It is unknown whether the STEC detected in coyote and river otter scat represents carriage or transitory colonization within these animals.

The common risk factor among the case-patients in this STEC O157 outbreak was exposure to a natural stream within a city park. After the outbreak was recognized, signs warning of bacterial contamination were posted along the stream. No further STEC O157 infections attributed to stream water exposure were reported.

Technical Appendix Detection of Shiga toxin–producing *Escherichia coli* in wildlife scat specimens along a stream implicated in outbreak among children, California, May 2016.
