# Supplementary material for: Contaminated Stream Water as Source for Escherichia coli O157 Illness in Children
Source: Emerg Infect Dis. 2017 Jul;23(7):1216–8. doi: 10.3201/eid2307.170226 (PMC5512484; doi:10.3201/eid2307.170226)
Supplement: Technical Appendix — Detection of Shiga toxin–producing Escherichia coli in wildlife scat specimens along a stream implicated in outbreak among children, California, May 2016. [file 17-0226-Techapp-s1.pdf]

Article DOI: https://dx.doi.org/10.3201/eid2307.170226

Contaminated Stream Water as Source for *Escherichia coli* O157 Illness in Children

Technical Appendix

Technical Appendix Table. Detection of Shiga toxin–producing *Escherichia coli* in wildlife scat specimens along a stream implicated in outbreak among children, California, May 2016

Scat source	Distance from exposure site	PCR	Culture isolation
Coyote	0.2 miles downstream	stx_1, stx_2 detected	Stx1, Stx2 *E. coli* O157
Deer 1	1.5 miles upstream	stx_1, stx_2 detected	Stx1, Stx2 *E. coli* O157
Deer 2	1.6 miles upstream	stx_2 detected	Stx2 *E. coli* non-O157
Deer 3	Exposure site	stx_2 detected	STEC not isolated
Deer 4	Exposure site	stx_2 detected	STEC not isolated
River otter	0.3 miles downstream	stx_2 detected	STEC not isolated
